# Bibliographical Notices, &c.

**Published:** 1845-10

**Authors:** 


### SURGICAL AND PRACTICAL OBSERVATIONS ON THE DISEASES OF THE HUMAN FOOT: WITH INSTRUCTIONS FOR THEIR TREATMENT. TO WHICH IS ADDED ADVICE ON THE MANAGEMENT OF THE HAND. By *John Eisenberg*. 4to. pp. 252. Renshaw, London, 1845.

THIS is a book on an attractive subject. We have seldom met with a person of either sex, however deeply absorbed in the serious pursuits of this world, who, if possessing small and well-turned feet, or white and neatly-shaped hands, were not proud to display and careful to preserve them, and everyone recollects the vain notion of our great and titled poet, of the size of hands as indicating birth, his own being aristocratically small.

John Eisenberg, who dedicates his work to Dr. Marshall Hall, seems to have felt the necessity of showing good cause for writing a book, and, like most authors, is desirous of impressing the public with the importance of his subject. Accordingly, after contrasting the neglect which the human teeth, and the diseases of horses formerly experienced with the attention now paid to these subjects by educated men, he expresses a hope that, before any great length of time has elapsed, Chiropodists will be admitted to their place in the social system, and that, as in the large towns of Germany, the staffs of our hospitals will be increased by dignified professors of an art, who, to the author's surprise, now rank, in this enlightened country, only as humble corn-cutters.

In a society consisting of persons either too fastidious or too indolent to cut their own corns, a necessity may exist for this class of operators, and we have no objection to their assuming the euphonious title of Chiropodists, and as such, *taking their proper place in the social system;* but we most strenuously resist any invasion of our province as Surgeons by any of these special professors, whether native or foreign, and, although our author takes some pains to convince his readers to the contrary, we must take the liberty of stating that every sensible surgeon perfectly understands the structure of corns and bunions, and their causes and treatment, as well as the management of some other small affections, such as the "growing-in of the nail into the flesh," as it is termed, warts and chilblains, which our author seems anxious to take out of our hands. It is really time to extinguish these pretensions, for if we give up to advertising Aurists and Oculists, Dentists, Chiropodists, Orthopædists, Skin-doctors, and Bath-proprietors, all they would claim, there will be very little left to the charge of the general Surgeon.

If a large and handsome volume can confer dignity on his subject our author has fully availed himself of the advantage. This book is of quarto size, printed in large type, with ample margins and handsomely bound in blue cloth with gilt-edged leaves. It is indeed, as the author no doubt intended, an elegant drawing-room book. We shall content ourselves with gleaning such practical remarks and hints as may seem likely to interest or to be useful to our readers.

The author does not coincide in the general opinion that corns are the result of pressure. He remarks :—

"It has been erroneously asserted that pressure upon the epidermis is the sole cause of corns; that its vessels, becoming injured and hypertrophied, throw out a larger quantity of lymph than is necessary; and that the consequence is the generation of layers, which become interwoven. Were pressure the sole cause of corns they would not be confined to so small a surface, they would necessarily embrace a much larger portion of the foot, and we should find the heel, where the greatest pressure exists, the most frequent seat of disease. Some persons are annoyed in a most extraordinary degree by corns, who are the most careful in the selection of their shoes, whilst many who are in the habit of wearing such tight shoes as to be almost incapable of walking, and who seem to wish to resemble, in their incapacity for movement, the Chinese ladies, are totally free from them; besides which, infants have been known to have striking excrescences. There is no doubt whatever that the pain and uneasiness attendant upon a corn are exacerbated by the pressure of a tight shoe, and that instantaneous relief attends a change; but this is easily explained. A new boot sometimes is said to have been the cause of the mischief, whereas the corn has not only long existed, but has formed for itself, in boots that have been worn, a cavity. We constantly find that those who live in towns are more subject to corns than those who live in the country, although the materials of which their boots and shoes are made are so much harder and rougher. It is not at all an uncommon thing for a person who has been afflicted with serious excrescences to lose them altogether whilst in the country, and again to be burthened with them on his return to London. The alteration of structure, wherever corns exist, is of the most decided character. No longer can it be considered as organized texture, for the laws of vitality have altogether ceased within its immediate range; vessels no longer circulate through it their normal fluid; nor does the rete mucosum furnish longer that mild lymph which lubricates the superior surface of the cutis vera, and the inferior surface of the epidermis, but in its place there is an exudation of a serous-like fluid, which rapidly hardens and thickens, layer accumulates upon layer, a corneous substance is formed, which gradually insinuates itself either amongst the muscular fibre, or the minute arterial vessels enter into the softer and spongier parts, assist in giving here and there vitality, and become the source of that exquisite pain which is often complained of by the sufferer." P. 15.

The preceding observations will make very little impression on our readers. The pathological remarks are too absurd to merit notice, and the character of the book will be understood when we state that the author magnifies the difficulties and dangers of removing corns by operation, and dwells on the disastrous and even fatal consequences of a badly-performed operation. On the subject of callosities, the author remarks:—

"It is a curious fact that those who ride on horse-back and are amongst the foremost in the chase, are strikingly liable to these affections. We have known men who are remarkable for their love of hunting, who have no other rural pursuit to which they are partial, who neither shoot nor fish, and who, when in town, take little but carriage exercise, suffer in a singular degree from hard, thickened, callous indurations around the heel, and they are not unfrequently exceedingly sensitive. The whole epidermis is in such cases unusually dry, ragged, and small papulæ in groups cover its surface; those who are advanced in years seem to be more subject to the rapid advance of these indurations, and they seldom can check them unless they have frequent recourse to the pediluvium of a high temperature, followed by rubbing the surface for some considerable length of time with a rough dry-towel; indeed many persons have found their only relief from following this plan systematically for a considerable length of time, and even if they have abandoned it, from the total absence of the callosity, they have found it to return and to become even more troublesome than before." P. 126.

The following is the plan recommended for the treatment of warts.

"The hydrochlorate of ammonia dissolved in water, and the hydrochlorate of lime are the most certain means of destroying them; the process, however, in both instances is very slow, and demands perseverance, for if discontinued before the proper time, no advantage is derived. The warts are to be kept constantly moist with the solution of one or other of these neutral salts, small folds of linen are to be soaked in it and applied at night, but this must be duly attended to. It must not be the practice for two or three days and then laid aside; it must be followed up for a month, at the end of which time it will be found that the excrescence no longer exists, and it will not again return. Success almost always follows upon this plan, and therefore it is the one I would urge as that upon which the greatest reliance can be placed. The most obstinate warts, that had baffled the attention of the most skilful, and that had re-appeared after other treatment, completely yielded to this; and since it has become under ordinary circumstances the plan I recommend to be pursued." P. 147.

Those who suffer from immoderate perspiration of the feet are recommended to use a solution of the chlorate of lime or of soda for its prevention, more especially if it be attended with any fætor, which sometimes occurs, but more generally as an indication of constitutional disorder than of local affection. The sponge, moistened with equal parts of camphor julep, of Mindererus' spirit, with a few drops of spirits of lavender, will often put a stop to the most disagreeable effluvium which arises.

The author treats chilblains on the feet with cold applications. He observes :—

"The production of cold, by the evaporation of ether, by the use of spirit, or the immediate application of ice, not only gives temporary relief, but permanent, if it be persevered in for some time ; compresses, dipped in cold lotions, should be unremittingly kept upon the part affected until the redness, heat and swelling have disappeared. The warm fomentations, such as decoctions of poppies, of chamomile flowers, of turnips, are occasionally successful, but I by no means recommend them, for I have been uniformly successful in the removal of chilblains in their inflammatory stage by means of cold lotions." P. 188.

We must here conclude our notice of this work. It is scarcely deserving of even the brief attention we have given to it. The object of the author, it is too obvious, was that of making a book to serve the purpose of advertising himself, and we must express our regret that a distinguished physician should have sanctioned such a proceeding, by permitting his name to appear on the first page of a work of so questionable a character.

---

THE COLD-WATER CURE, ITS USE AND MISUSE EXAMINED. By *Herbert Mayo*, M.D. F.R.S. 12mo. pp. 85. London, 1845.

HOWEVER pleased at hearing a medical brother's shattered health has undergone restoration, we can but regret that the same process has converted the *ci-devant* respectable surgeon of the Middlesex Hospital into the *doctor* of a hydropathic establishment. Any alliance of this kind inflicts disgrace upon the profession by imparting undue sanction to quackery. It is true Dr. Mayo is not an implicit follower of the charlatan Priesnitz, and notices not a few instances of the evil resulting from indiscriminate employment of the various appliances. He believes that, by variations in the mode of application, very various results are produced, and that, to appreciate these, preliminary medical information is required. He desires to employ hydropathy co-operatively with other medical means, not exclusively.

"I do not adopt and use it without modifications which Priesnitz would repudiate as hostile to the spirit of his method. But I take its elements and employ them my own way. Perhaps, if the prescribed routine had suited my own case, I might have been misled by it. But my own case was too serious, and could not be cured by the system with its errors; it happened to require and admit of a part only of the routine treatment; and in following this view, and looking to see how much each individual case of serious disease requires, the system has disappeared, and in the place of the cold-water cure, I discern only a more extended and scientific use of cold-bathing."

Doubtless Priesnitz would assert that if the author had entirely, instead of partially, followed his system, he would have been by this time completely cured, instead of being, as he is, only so in part. Having been for some years a victim of rheumatic gout, he repaired, at the advice of Sir J. Clark, to the hydropathists in 1842, and after douching, stewing, and sweating for a couple of years, feels himself much better, and thus winds up his account of his case, which we suppose is set down as a *cure!*

"In September I found myself almost suddenly much better; my feet and ankles, which up to this time had regularly by the evening become large and heavy, ceased to swell, and were hardly larger at night than in the morning; my knees at the same time became reduced in size, and I could stand every day, and most days could walk a few steps. *As I expected, I have since fallen back a little;* but I can now always stand without support on both legs, and I am confident that *next Summer I shall make the remaining step of walking.* In general strength I palpably improve every quarter of a year; the rheumatism burns out more slowly."

The various means at the command of the hydropathist may, according to our author, be made to operate in four different modes. 1. The *tonic* course, consisting of cold bathing, friction, and exercise, and cold water drank in moderation as a "*stomach bath.*" This is applicable to cases of general debility, feeble circulation, deficient innervation (as in hysteria and mental depression, certain forms of palsy, &c.), threatening or incipient scrofula, muscular rheumatism, and regular gout in certain habits. 2. The *reductive* course, consisting in the induction of profuse sweating, "with just enough cold bathing afterwards to prevent the debilitating effects." This, according to Mr. Mayo, is seldom indicated, and he gives various examples of the evil resulting from its employment. 3. The *alterative* course. This is applicable to many cases, and consists in antagonizing the sweating and cold bathing processes, so as not on the one hand to reduce, or on the other to stimulate the patient; but, to give tone to his system by exciting a moderate action of the skin. Over-taxing of the bodily or mental powers, general disorder of the system, threatening head-disease, dyspepsia, irregular gout, or chronic rheumatism are cases for its use. 4. The *sedative* course consists in maintaining a prolonged application of cold water to prevent re-action taking place, and is applicable to cases of fever, inflammation, spasm, mental excitement, &c.

The conclusion we arrive at from the perusal of this pamphlet is, that there are various cases of dyspepsia and broken health, proceeding from gormandizing, idleness, anxiety, &c. which are greatly to be benefited by the change of air and scene which has been so long and so frequently recommended for them; and that, in some of these, the cold-water discipline might prove an useful auxiliary—provided it were administered under medical inspection. But we believe the present rage for its adoption is dangerous in the extreme, and that while fancied ailments have been by its agency frequently converted into real diseases, and slight ones have been frequently rendered serious, not a few lives have been sacrificed at this new shrine of empiricism. The vast majority of diseases to which the system is applied are more easily and more safely curable without it; and if the rough practices it sanctions have worked a happy revolution in the constitutions of some old or young debauchees (which class constitute a large share of Priesnitz' patients), more delicate frames, which, under judicious treatment, might often have been renovated, have succumbed to them. Again, therefore, we regret that any professional man, so well-known by his former labours as Mr. Mayo, should have given his sanction to practices so questionable in their therapeutical tendencies.

It must be a bad book that furnishes no useful hints; so, in parting with this one, we may inquire of our reader if he ever suffers from headache, defective digestion, or a slow action of the bowels. If so, let him seat his nether-end in a tub of cold water from five to thirty minutes, and the benefit he will thence derive, especially if he can command a rapid renewal of the fluid during the *seance*, will be surprisingly great. For ourselves, we are so liable to headache and weariness in pursuing some portion of our critical duties, that we feel grateful to the author for his panacea, and only regret that we were not in possession of it a little earlier, as we should like to have tried the experiment of reviewing "The Cold-water Cure" in a "Sitz-bath."

---

CONTRIBUTIONS TO THE MEDICAL HISTORY AND TREATMENT OF SEXUAL DISEASES. By *John Hey Robertson*, M.D. Octavo, pp. 80. Glasgow, 1845.

THE first part of this *brochure*, treating of M. Ricord's practice in the Venereal Hospitals of Paris, was published by the author in a local journal as far back as 1833; and if he thought it worth while to reproduce it, he should have corrected its loose, rambling style, quite inexcusable in a re-print. Dr. Robertson is an enthusiastic admirer of the practice of *inoculation*, but yet inconsistently enough never employs it. He says—

"We have no means as yet of making an accurate diagnosis but by inoculation—but, since it can do the patients no harm, and may do them a great deal of good, there can be no reason why they should not submit to this very simple, and not in the least painful, process, equally for the surgeon's assistance, the benefit of science, and their own safety. (Notwithstanding this, I have never yet in this country done such a thing, *or even proposed it!*")

M. Ricord, however, inoculates in three places on the inside of each thigh in every case of suspicious sore or gonorrhœa, repeating the operation again and again if it fail. The healing of the original chancre takes place more quickly than when this practice is not adopted, the inoculated one also disappearing in a day or two afterwards. Sores on the glans or pudenda, which will not produce their like on inoculation, and which are very common, are termed *Herpetic* by M. Ricord. The *outrance* to which M. R. carries his opinions concerning the diagnostic value of inoculation is exhibited in one case, although not cited by the author to such end. A woman with sores was inoculated no less than ten times and in 60 places, without any effect whatever. The Professor, somewhat staggered at this at first, afterwards solved the difficulty by declaring "that he regarded this as a very extraordinary specimen of true syphilitic disease, as no less than a primary sore having passed into a secondary, without the constitutional affection!" M. Ricord having observed that *gonorrhœa* sometimes communicates chancre by inoculation, believes that cases of this disease, which are followed by secondary symptoms, are always those in which a chancre has existed in the urethra; and Dr. Robertson says that, before he was aware of this opinion, he had remarked that many gonorrhœal patients to whom he gave *pil. hyd.* as an alterative, but which must really have acted as an anti-syphilitic, got well very rapidly.

In his treatment of *Syphilis*, M. Ricord endeavours, if possible, to heal the sores without mercury, believing that secondary symptoms are then of less likely occurrence. Dr. Robertson, however, here seems to have lost faith in his instructor, for he says, "Indeed, notwithstanding this gentleman's expressed opinions, it is doubtful whether in this instance he believes them himself; for, in the course of some disputations with him one day, I suddenly put the question to him, 'How would he do in his own case?' The reply was *characteristic* (!) 'Ah! c'est une autre affaire.'" M. Ricord applies the *nitrate of silver* to the sore, giving mere ptisans the while. If, in a week or two, he finds no sign of healing, he prescribes the *proto-ioduret of mercury* in small doses, never continuing it long enough to affect the mouth—in older cases adding sarsaparilla, and ordering warm-baths.

For the treatment of *Gonorrhœa*, M. Ricord makes much use of injections, *lead* being the substance he prefers. He does not allow that stricture, hernia humoralis, &c. ever result from their employment. Dr. Robertson believes that *cubebs* seldom do any good, and sometimes cause violent irritation, requiring free leeching. Of *copaiba* he has not a much better opinion; and has found *ol. terebinth.* given in small doses the best medicine for oldish cases. It may be combined with *liquor potassæ*, or, if it purges, with *opium*. He seldom uses injections, and considers a weak solution of *arg. nitr.* the best, and *cupri sulph.* the worst of these.

Dr. Robertson regrets that the *Speculum*, so constantly used in France, is so little employed here. It reveals the fact stated by Ricord.

"That, with hardly an exception, every woman who has gonorrhœa or whites, will be found to have abrasion, ulceration, herpes, or some other affection of the neck of the womb, or superior part of the vagina—generally the former—and that, though the gonorrhœa or whites may be removed, this disease found so far beyond the reach of the unassisted eye, and though it be unable to produce any sore by inoculation, yet possesses the property of producing discharge in man by connexion."

When the discharge is found to proceed from the os uteri, an iodine injection (Træ ʒj. ad aq. ℥iv.) is to be employed. In mere *herpes*, not communicable by inoculation, rest, diet, and cleanliness suffice; but when there is abrasion or ulceration of the cervix, the red oxide of mercury, dissolved in nitric acid, forms the best application.

The second chapter of the work contains an account of the maltreatment of a case of secondary syphilis for one of rheumatism by the hydropathists, the patient eventually losing an arm in consequence of the formation of abscesses in the wrist. The author is however in error, in supposing that the water-doctors would have hesitated undertaking the case had they known it to be one of syphilis. Old pox cases form a large proportion of the Silesian peasant's *clientelle*.

The last paper, detailing two or three cases of spermatorrhea resulting from onanism, might as well have been omitted, for any novelty it contains; and indeed we think the author might have been well contented with the medium which any of the weekly periodicals would have furnished him for the publication of the entire pamphlet. But then he would not have written his BOOK.

I. THE RETROSPECT OF PRACTICAL MEDICINE AND SURGERY, &c. Edited by *W. Braithwaite*. Vol. XI. January—June, 1845. 8vo. pp. 335.

II. THE HALF-YEARLY ABSTRACT OF THE MEDICAL SCIENCES, &c. Edited by *W. H. Ranking*, M.D. &c. Vol. I. January—June, 1845. 8vo. pp. 391.

MUCH is done in the present day to render knowledge easy of acquisition, in Medicine as in other departments of science and literature. There are numerous weekly journals, recording the passing events of the day, containing too the reports of some admirable lectures, and stored with many useful communications from practitioners in all parts of the world. Besides these, there are not a few monthlies, whose communications aim to be of a higher and more elaborate character; and several quarterlies, which are devoted chiefly to the review and analysis of the more important works that issue from the press. As if these were not enough, we have now, in this country, two semestral periodicals, which profess to contain all the valuable pith and substance of the rest, skimming the cream from their surface, and selecting (what the editors consider to be) the wheat from the chaff. In this manner it is believed that the concentrated essence of the preceding six months' experience is expressed and obtained; the reader being thereby saved the trouble of gathering the information and distilling it for himself; on the same principle—to use a professional illustration—as the pharmaceutist finds it a most convenient method of preparing his " aquæ distillatæ" by rubbing up a few drops of an essential oil with water, in place of the more troublesome and tedious method recommended in the Pharmacopœia. But the question comes to be, is the medicament as good, when obtained in the one as in the other way? Is the aromatic and active principle in the first preparation equably diffused and incorporated with the mass of the liquid throughout? or is it not apt after a time to float upon the surface, the water beneath being then vapid and worthless? We must leave our pharmaceutic brethren to answer the question, and shall not presume to decide it ourselves. But of this we feel pretty well assured in reference to professional knowledge, that whatever is got up very quickly is seldom retained very long, or applied very skilfully. It has been said that there is no *royal* road to learning; is it not equally true that there is no *popular* one? This remark holds especially true in the study of medicine. Much that seems easy on paper will be found difficult of application in practice; and the physician will often feel himself perplexed by the very multitude of the really useful hints and recommendations, that are continually making their appearance in the pages of our journals, unless he has some leading principles to direct him, and has made himself thoroughly acquainted with the nature and real cause of diseases, as well as with their more obvious symptoms and the remedies that are usually employed for their relief. It is for this reason that it has always seemed to us that the careful study of two or three standard works, in the course of each twelve months, would be infinitely more serviceable in the long run to most medical men, than the skimming perusal of all the encyclographies and periodical digests that are published at home and abroad. The one course of reading will make a *sound*, the other may make a *ready*, practitioner.

Of the two publications, whose titles are prefixed to this notice, the first one has been before the profession for the last five or six years, and has met with very considerable encouragement. But Mr. Braithwaite, it would seem, is now not to have the field to himself; a competitor has made his appearance, and—rather strange to say—puts forth his claims to public favour as if his project was an entirely new one; he does not so much as deign to mention even the name of his predecessor. Is this quite fair and honourable? Why should Dr. Ranking, if confident of the superiority of his work, not boldly and manfully challenge a comparison with, not the *Jahrbuchers* of Germany and the *Encyclo-*

*graphies* of France and Belgium, to which he refers as his models, but with the *Retrospect* of a brother practitioner, not a hundred miles off? If merit is due to any one, for having proposed and carried into effect the idea of having a half-yearly abstract of the progress of medical science, it unquestionably belongs to Mr. Braithwaite: there can be no doubt, upon this point. Dr. Ranking's work, indeed, is one of higher pretensions than that of his predecessor, professing, as it does, to be not only a repertory of the most interesting communications in other journals, but also a review of new works; for he tells us in his Preface that "not only is every periodical of note published in Great Britain, America, France, and Germany subscribed for and personally consulted, but every standard publication and monograph which can be obtained is analysed, as it may come to hand." The *review* department, as a matter of course, is miserably jejune; on the one hand, there is no notice whatsoever of several of the ablest books that have been published, during the period embraced; and, on the other, the reader is introduced to an acquaintance with some works that have been perfectly well known to the reader for the preceding three or four months. We have an example of this in the case of Dr. Golding Bird's recent treatise on Urinary Deposits. While Mr. Braithwaite very deliberately takes (with acknowledgment) about ten pages from *our* review of this valuable work—in the Number of the Medico-Chirurgical for April last,—Dr. Ranking proceeds in July to give a condensed analysis of its contents, being "convinced," he says, "that the brief summary, which we shall give, cannot fail to produce the desire for a perusal of the original work."

By far the best part of the Doctor's work is the latter half, containing semestral Reports on the progress of Medicine, Surgery, Midwifery, Anatomy and Physiology, Physiological and Pathological Chemistry, and of Forensic Medicine. Of these, the Report on Anatomy by an anonymous hand, and that on Chemistry by Dr. Day, are the most complete and elaborate. The selection of extracts, in the first half, is unquestionably not equal in point of value to that in Mr. Braithwaite's work. Would it not be better for Dr. Ranking to leave this department entirely to his predecessor, and devote his talents to the redaction of comprehensive reports on the different branches of medical science?—a task that would be very acceptable, and gratefully received, we doubt not, by the profession. Whether this hint be acted upon or not, we can have no hesitation in saying that the "Retrospect" will be the most useful to the practitioner, and the "Abstract" to the literary student.

---

THE IRISH WATERING PLACES, THEIR CLIMATE, SCENERY, AND ACCOMMODATIONS, &c. By *Alex. Knox,* M.D. 8vo. p. 336. Dublin, 1845.

DR. KNOX was recommended, in consequence of protracted illness, to try the effects of continued travelling and change of scene. Instead of following the multitude to visit the spas of Germany, or the azure shores of the Mediterranean, he resolved to make a prolonged tour through his own deeply-interesting and romantic country. The result of his travels is the present volume, which we can confidently recommend to the reader as alike amusing and instructive. Dr. Knox has done good service to his native land by drawing public attention to its many valuable resources, in a hygienic and sanatory point of view. We trust that his meritorious labours will be appreciated, as they deserve, not only by his own fellow-countrymen, but by the profession generally throughout the kingdom, as an ably-executed design "to do for Ireland what has already been so amply accomplished for British and Continental Spas." Dr. Knox has received much assistance, in the execution of his work, from the communications of the medical men resident in the different localities which he visited. The most valuable of these is an elaborate report on the climate of Cove (near Cork) from Dr. Scott, who has long enjoyed an extensive practice there. It is a most desirable locality for consumptive invalids; perhaps the very best in the United Kingdom. The report of Dr. Scott is well worthy of general perusal; we should gladly have extracted a large portion of it, had space permitted. We must therefore refer our readers, for information upon this and other points connected with the Irish Watering-places, to our author's work, which, besides strictly professional matters, contains a good many interesting geological and botanical details.

---

### IS RAILWAY TRAVELLING CONDUCIVE TO APOPLEXY? By *J. C. Badeley*, M.D. Small 8vo, pp. 16. London, Smith, Elder, and Co. 1845.

HUSKISSON seems to have had a clear insight into the future, when he predicted that "England will ultimately resemble a large gridiron." The gridiron-mania had not then risen to such a pitch as it has now attained, and the apprehension of apoplexy is added to the long catalogue of broken bones and other dangers attendant on rail-road travelling.

We believe there are but two or three well-authenticated instances of apoplexy *taking place* in the train. Mr. Locksley had disease of the heart, and was long threatened with cerebral affection before his fatal journey with Sir Henry Halford. Lord Canterbury was also of a full plethoric habit for years before his death in the train. From long observation, and no small experience in rail-road travelling, we are convinced that there is little or nothing in the physiological action of the train either to predispose or to excite apoplexy. Gestation, or the passive exercise of the carriage, has a tendency to equalize the circulation, determine to the surface, promote the insensible perspiration, and consequently to dispel rather than promote local congestions, whether about the head or other parts. This is the opinion, clearly, of Dr. Badeley, whose sensible little brochure will tend to dispel the unfounded fears of railway travellers.

"The question is, Whether there is any thing in the act of being smoothly drawn along at a rapid rate that is calculated to cause it? We hear of death occurring from apoplexy 'in the stilly hour' of night, as well as in the bustle of the daily world—under circumstances of calm tranquillity, as well as of mental agitation or bodily exertion; and it would be difficult to prove that the crisis would not have occurred had the sufferer been in his study, or in his bed, instead of enjoying the itinerant luxury of a first-class carriage!" P. 10.

There is one precaution, however, which we would recommend to railway travellers who have any disposition to vertigo or other affection of the head—it is to avoid looking at the *near objects* on the road-sides. These appear to fly along at so rapid a rate as to cause some degree of giddiness in particular constitutions, and the cause ought to be avoided by people of that description. We hope to see something of still more importance from the pen of the accomplished author of the present brochure.

---

### REGISTRATION OF THE CAUSES OF DEATH.

WE have received from the Registrar-General a book of blank forms for medical Certificates of the cause of Death, and also a circular letter addressed by him to all legally-qualified practitioners in England, requesting of them to use the printed form (in place of a written statement,) upon all occasions, filling up the blank spaces upon it to the best of their knowledge, so that as accurate a return as possible may be made of the most important circumstances connected with the death of each individual. Each Registrar is instructed to supply every medical man in his district with one of these books, which is similar to a banker's checque book, and is exceedingly well suited for the purpose. By using it, much trouble is avoided, and far greater accuracy is insured. We therefore trust that every member of our profession throughout England will at once apply to the proper quarter for one of these books, and if he is not already provided with the "Statistical Nosology,"—that has been recommended for general adoption—he has only to send a written application to the General Register Office, Somerset House, and a copy will be forwarded free of expense. The Registrar-General deserves very great praise for the zeal and liberality that he has uniformly shewn in the various arrangements of his office; and it only requires the hearty co-operation of the medical profession in supplying accurate information as to the cause of death, to collect together a vast amount of most interesting and useful knowledge on the important subject of Medical Statistics.

---

### Extracts from the Quarterly Medical and Surgical Journal for the North-Western Provinces—Fever, Cholera, Dysentery, &c.

In our last number, we acknowledged the receipt of the first four numbers of this new Journal, published in the very heart of India, being printed at Delhi by Kunniah Lall. The Editor's name is not given; but the great merit of the undertaking is obviously due to Dr. W. L. McGregor, surgeon of the 1st Bengal European Light Infantry. The first number is almost entirely written by him, and by far the largest part of the others is also from his pen. Each successive number is more valuable than its predecessor; and we are glad to find in the last one a very respectable list of subscribers' names, among the medical men throughout India.

The opening article of the first number, *on the close connexion between Fever*—the intermittent, and more particularly the bilious remittent, forms in tropical countries—*Cholera* and *Dysentery*,* contains many interesting and truly practical remarks. All these diseases are produced by the same morbific agent, Malaria; in all, the proximate or essential cause appears to be "a congestion of the biliary organs" (usually accompanied with a distended state of the gall-bladder), the result of a general depression of the entire nervous power; and they should all be treated upon the same principles.

The stage of collapse in Cholera may be regarded as the cold or congestive stage (in its most intense degree) of a Fever; and the great object of the physician ought therefore to be to bring on the subsequent stages of febrile reaction and of perspiration, as necessary to effect the recovery. Nature herself generally succeeds in removing the internal congestion at the commencement of a Fever, by the rigors that then take place; but in Cholera, the prostration is often so great, that she sinks under it without effecting the desired end.

In Dysentery, there is never the same degree of internal sanguineous congestion that we observe in Cholera and Fever; its chief danger consisting in the local inflammation of the bowels that is so generally present. Nevertheless, there is every reason to believe that, in the early stage of this disease also, the biliary organs are much engorged, and that the gall-bladder is distended with dark and viscid bile.

Nothing so effectually serves to counteract and remove visceral congestion as the action of Vomiting; and the medical man must therefore be most careful that he does not use any means to arrest too quickly this (so frequent) medicative effort of Nature, until the stomach be effectually cleansed, and the peripheral

---

* Dr. M. adds *Rheumatism* to the list; but, as our remarks will apply chiefly to the others, we shall leave it out of our consideration at present.

circulation is in some degree restored. A vast deal of harm has been done in Cholera, more especially, by the injudicious administration of large doses of opium, and other sedatives to stop at once the vomiting and purging, in the early stage of the disease.

Dr. McGregor remarks: "In a recent case of Fever, when there is no headache, an emetic will often remove the congestion; when more severe, with headache and great heat of scalp, I give the following pill:

℞. Ol. Crotonis Tiglii, gtt. v.
Extr. Hyosciami, gr. v.

If bile is present in the stomach, vomiting is generally produced;* and by this effect, and the free evacuation of the bowels, all the febrile symptoms subside, and apyrexia is obtained; in short, Congestion, or the essential cause, is removed. The next object is to strengthen the nervous system, without which the congestion will again occur, and the danger to the patient be increased; 10 grains of quinine are then to be administered, and, if the bowels have not been freely opened, the compound quinine mixture is to be given every hour, in addition to the dose of quinine; this mixture is as follows:—

℞. Sulph. quininæ, gr. xij.

Acid sulph. dil., dr. ½.

Sulph. magnesiæ, oz. ½.

Aquæ, ℔ ½. M.

"An ounce every hour will answer, and this contains half a grain of quinine and half a drachm of salts; the latter may be omitted where the bowels have been freely purged by the croton, which is generally the case." P. 11.

Bleeding, blisters, turpentine enemata, calomel, &c., are required in particular cases, to remove particular symptoms.

*Dr. McGregor's Treatment of Cholera.*

Our author's favourite remedy—one which, he assures us, has scarcely ever failed in curing the disease, when timely and rightly administered—is Croton oil and (hill) opium; five drops of the former, and from three to five grains of the latter, in each dose.

He says: "If no blood can be obtained, I give the following draught immediately:—

℞. Croton, gtt. v.
Tinct. Hyosc., dr. j.
Opii (hill), gr. v. M.
ut fiat haustus.

"If the spasms remain, and free vomiting does not succeed, the following pills are administered:—

℞. Opii (hill), gr. iij.

Ol. Croton, gtt. v. M.

ut fiat pil.

"If the symptoms continue, the pill is to be repeated (the mass may be made into two with bread crumb) until they subside, and *free vomiting* ensue, when the cold clammy skin becomes warm and moist, and the tongue and expired air participate in the healthy change in general; nine grains of opium and fifteen drops of the oil, in repeated doses, will produce these effects; but in one case, I gave 18 grains of opium and 27 drops of croton oil before the disease yielded, and it is satisfactory to learn, that in every case the treatment has been successful, where I have had the sole management of the case. To prevent a relapse, the use of quinine is advisable." P. 14.

Dr. McGregor, in a subsequent paper, relates several cases of Cholera suc-

---

* Large doses of Croton oil almost always produce vomiting.

cessfully treated in this manner. In one, the patient took 45 drops of croton oil, and 39 grains of (hill) opium in the course of five hours!—he recovered. Allusion is made to another case, in which 35 drops of the oil were given in the course of one hour! As we have already said, this medicine acts as a powerful anti-congestive, by the vomiting and purging it produces;* while the opium serves to soothe the nervous system. Along with the treatment now recommended, saline or turpentine enemata, and blisters or turpentine epithems may be freely used.

*Dr. Jephson's Treatment of Cholera.*

This gentleman, in his account of the cholera at Sukkur in 1840, tells us that, after trying ineffectually the ordinary remedies—stimulants, calomel and opium, sinapisms, &c.—"he determined to pursue the same treatment as is usually followed in Ague;" being convinced that the two diseases were strictly analogous alike in their symptoms and origin. Acting upon this impression, he had recourse to the exhibition of Emetics and neutral salts, "modified and combined with opium and stimulants in some of the stages." The results were very favourable. His usual formula was a mixture, consisting of an ounce of Epsom Salts, two grains of Tartar Emetic, and eight ounces of water: the dose one ounce every half-hour. After the third or fourth dose, the vomiting and purging often ceased. In some cases, the use of this mixture was preceded with an emetic of Ipecacuan. One or two large doses of Calomel, effervescing draughts, sinapisms, and turpentine epithems to the abdomen, were the adjuvants that seemed to be most useful: Quinine was generally given after the cholera symptoms had subsided. Opium appeared invariably to do mischief in the long run: it seemed to increase the tendency to a relapse. Dr. Jephson remarks, in reference to the *modus operandi* of his plan of treatment, that "it is not the simple effect of an emetic which is beneficial, but it is the *sedative* influence of the saline tartar-emetic to which I attribute the benefit derived from its use."†

Dr. Anderson has related two cases in which very decided benefit was obtained from the use of saline enemata—composed of from half to one ounce of common salt, and one drachm of carbonate of soda in a pint of warm water—to be repeated every hour or oftener, until some decided effect is produced.

*On the Use of Nitrate of Silver in Chronic Dysentery.*

"The employment of lunar caustic as a component and active ingredient in

---

* "In this disorder (Cholera), vomiting is described as one of the symptoms; the action is not, however, vomiting, but an ineffectual attempt at the latter; and when the stomach is able to act freely, and remove its contents, the symptoms of Cholera disappear, and the congestion of the biliary system, or, in other terms, the essential cause of the disease is removed, and instead of the white congee stools, totally divested of bile, or fæculent matter, those containing both are speedily voided, and the body is thus restored to a state of health, and that from a disease which kills in a few hours!" P. 117.

† In illustration of this remark of Dr. Jephson as to the *sedative* influence of the tartar-emetic, we may adduce the following paragraph from one of Dr. McGregor's communications:—

. . . "Finding, in the case of cholera at Loodiannah, that the irritability of the stomach was only allayed effectually by six grains of hill opium, and the same quantity of tartar-emetic, he was ordered this dose; and at 1 P. M. the report stated, that he was asleep, having previously been purged, voiding a *dark stool;* this was the first change in the alvine dejections. The stomach, after this, became less irritable, and he retained sago and a portion of wine which he used as food, while the quinine and opium were given internally." P. 249.

enemata is well known, and its internal use has, likewise, been often witnessed in chronic dysentery, particularly the mercurial form, but the quantities employed in both ways have been generally too small. Combined with half a drachm of opium in the quantity of a scruple it forms a powerful and efficacious means of lessening one of the most common and painful symptoms in chronic dysentery, namely, tenesmus with pain in the rectum and verge of the anus. In such cases any large quantity of fluid will not be retained, but with two ounces of mucilage or milk this enema is often kept for twelve hours. Given internally it is advisable to combine the lunar caustic with opium, and the form of pill I generally employ is the following:—

℞. *Opii, gr.* xij.

*Nit. Argenti, gr.* ij.

*Pulv. Ipecac. gr.* vj.

*Ol. Caryophilli gtt.* vj.

Divide in Pil. vj.—One of these is to be given every second hour, and the effect is often wonderful; but the action of such a substance must be carefully watched, particularly on the stomach, though its effects on this organ are less marked than that of sulphas cupri, or sugar of lead, while its action on ulcers must be much more beneficial."

*On the Use of the Spleen in Disease.*

From a short paper on this subject by Dr. McGregor, we extract the following passage:—

"It is, therefore, in disease that we must look for the use of the Spleen, and it has been proved that, in a state of health, an animal can exist without it; while, in great diseases, such as *fevers*, it becomes an organ of the utmost importance in preventing congestion of vital parts, and it may be doubtful whether cases, which we look upon as slight in the form of plain *intermittents*, might not often terminate fatally, were it not for the existence of the spleen; such fevers require but little of our aid during a first paroxysm. Miasma, acting on the nervous system and lowering its tone, produces congestion; nature, to remove this, sets up the cold stage, and the re-action or febrile state causes a disturbance in the circulation which is prevented from causing fatal congestion and effusion within the skull, by the blood being accumulated in the spleen.

"This leads to an easy solution of the use of the lancet in both the cold and hot stages of Fever; in the former, it removes a portion of blood, and enables the heart to act on the remainder efficiently, so as at once to remove the congestion and prevent a paroxysm altogether; in the hot stage again, bleeding prevents the congestion or accumulation of blood in vital organs, such as the brain, liver and lungs. An *emetic* given early is often useful in removing the congestion, and thus preventing a paroxysm which is only set up for its removal; and to the action of *croton oil* on the stomach and bowels is to be attributed the beneficial effect following its exhibition in fever, cholera, &c."

*On the Medicinal Effects of Tobacco.*

Dr. McGregor is, on the whole, favourable to the moderate use of smoking in a hot climate, provided the person does not give way to the filthy, and quite unnecessary, habit of spitting. According to his observations, it is unquestionably a prophylactic, to a certain extent, of the malaria of Fevers and other infectious diseases. A patient can hardly be salivated, if he continues to smoke; a circumstance that clearly shows how powerfully Tobacco counteracts the effects of Mercury upon the system. Whether it exerts a similar influence on other active drugs, such as Iodine, Digitalis, &c. we do not know; but it seems likely. During the rainy season, flies are apt to deposit their ova on blistered and ulcerated surfaces, and numbers of larvæ or maggots are thus produced. The best means of destroying them is by covering the abraded surface with tobacco-leaves moistened with water. In a curious case, where the larvæ appeared to occupy the *antrum maxillare,* Dr. M. found a decoction of Tobacco-leaves the only effectual means of removing them.—Moderate smoking of tobacco may be a useful preventive of Phthisis, as it unquestionably is of other pulmonic diseases. M. Simeon has recently noticed the unusual degree of immunity from Phthisical disease among the manufacturers of tobacco in France. In addition to smoking, the leaf might be applied to the chest.—The sleeplessness in Fever will sometimes be most effectually relieved by applying a moistened tobacco-leaf to the blistered surface of the scalp.

---

Memoir on the Field Carriage of Sick and Wounded Soldiers in the Bengal Army. By *J. S. Login,* M.D. Surgeon to the British Residency at Lucknow, Superintendent of H. M. the King of Oude's Hospital, late Surgeon to the Commander-in-Chief in India, &c. &c. Printed at H. M. the King of Oude's Lithographic Press. Lucknow, 1844.

Dr. Login's suggestions for the improvement of the field-carriage of sick and wounded soldiers are worthy of serious notice on the part of the Indian authorities, at home and abroad.

We can assure them of this truth, on no narrow view, and on no small amount of personal experience. We say it without one feeling of unkindness, that all military men, but especially those who have been long at the desk, are, beyond any other set of men, wedded to system. We have served long enough to know the value of systematic arrangement in the conduct of an army:—we, therefore, honour system. It is against the abuse of system that we would appeal. We mean the abuse of it resulting from ignorance, inattention, and inexperience.

We have attentively perused the Memoir of Dr. Login, and we find much in it that ought to recommend it to the military authorities, and especially to those authorities who may be in superintendence over the Medical Department of the Army. Of late years we have heard much of inertness and inefficiency in the said superintendence; but we dare say that Sir Henry Hardinge has looked well to this business, and that, to stand the oldest man on the list, will not any longer be received as the exclusive Patent to the Medical Staff.

What would become of an army whose Staff were chosen on the seniority principle? Every one can answer such a question:—yet, not all the staff of an army is more essential to its welfare, than the Medical Staff to the Medical Department of an Army. "Everywhere, and for all things, there must be a head," says Napoleon—(he does not say *heads*). We hope to hear better things of the Heads of the Medical Departments of India. But, to return to Dr. Login.

His Memoir exhibits the cumbrous, costly, and inefficient state of the field carriage for the sick and wounded, now in use in Bengal. He proposes to substitute another which, he says, is light, easy of carriage, more comfortable to the soldier, and greatly cheaper.

Dr. Login has shewn character, energy, and knowledge of his business in the field. He may be assured that, sooner or later, exertions like the present will receive their reward. Let him go on and prosper.

---

### Empyema with Purulent Expectoration—Recovery.

In the Fourth Number of the "British American Journal of Medical and Physical Science," edited by Dr. Archibald Hall, and published in Montreal, there is a valuable contribution by Dr. Macdonnel on the Diagnosis of some Thoracic diseases. He relates a most interesting case of Empyema accompanied with copious purulent expectoration, although there were no signs of any abscess in the lungs; and in which the effused fluid was gradually absorbed by the efforts of nature, without any operation.

The chief object, which Dr. M. has in view, is to establish the truth of the following position.

" *That purulent expectoration in Empyema, though attended by a quick pulse, sweating, emaciation and other hectic symptoms, is not indicative of tubercular or pneumonic abscess, unless accompanied by unequivocal physical signs of these lesions; but, on the contrary, it is to be regarded as the consequence of an effort of the constitution to get rid of a large collection of matter by one of the ordinary emunctories.*"

In the case related by Dr. M., the patient was a girl 15 years of age, in whom the symptoms of thoracic effusion on the left side came on in consequence of a neglected cold. The sputa were at first frothy, but soon exhibited a purulent appearance, and had a very offensive odour. The breath too was offensive, especially after a fit of coughing. On the fourth day (March 9th) after admission into the hospital (three months or so from the commencement of the pleuritic symptoms), " the quantity of pus expectorated amounted to about six ozs. in 24 hours; it was homogeneous and unmixed with mucus; its odour varied, at one time being very fætid, at another it was nearly without any smell; its colour was usually yellow, with a shade of light green." It deserves notice that the dyspnœa was very inconsiderable when the patient lay upon the affected side (indeed she could not lie in any other position); nor did her countenance betray much anxiety. Her appetite too was pretty good; but she was apt to feel sick after each act of expectoration, in consequence of the fætor of the sputa. The treatment, that had been pursued, consisted at first in cupping and blistering the affected side, and giving frequently-repeated doses of the *muriate of Ammonia* and *Digitalis:* subsequently, small doses of *pil. Hydrarg.* and of the *potass. hydriodat.* were ordered. In the course of a short time, although the sputa continued to be purulent and fætid, they were certainly not so copious, and moreover there was a larger admixture of mucus with them. The size of the left side of the chest became decidedly less upon measurement, and the general health of the patient was improved. On the 30th of March, " a loud friction-sound, having all the character of the *leather-creak*" was distinctly perceptible, alike to hand and to the ear, about the root of the lung. The recovery in this instance was most complete; for over every part of both lungs, the sound elicited by percussion eventually became clear; and the respiratory murmur was everywhere loud and pure. There was not the slightest reason to suspect the existence of any tubercles in either lung.

The following remarks by Dr. Macdonnel, on the peculiar character of the Sputa in Empyema, are worthy of particular notice.

" The fætor of the expectoration, and its occasionally bad quality, have been so frequently observed in cases of this disease cured by the vicarious elimination of the pus from the bronchial tubes, that we are naturally led to inquire into the cause of the phenomenon. To me it appears explicable by the fact, that in such cases we have a quantity of pus and air occupying the minute tubes and air-cells, *and having but an imperfect communication with the external atmosphere, owing to the larger tubes being nearly obliterated by the compression to which the lung is subjected by the fluid of the empyema,* and in this way they act chemically on each other, and produce a decomposition, giving rise to the intolerable odour, which both the pus and the expired air soon acquire. In fact, the same phenomena are observed in these cases as in an ordinary abscess, the matter of which may be healthy and odourless on its being opened, but soon becomes altered in these respects when air enters the sac and acts upon its contents, which then become bad in quality and offensive in odour. This view is borne out by what was noticed in Mr. M'Cullagh's case, viz. that the breath was not fætid during ordinary expiration, but became so immediately after coughing, by which the air *pent up in the remote tubes was expelled,* whilst that taken in, during ordinary inspiration, was exhaled devoid of odour."

There was a peculiar feature observed by Dr. M. in his patient, and which has not, as far as we know, been noticed in any similar case before;—viz. the presence of a loud *bruit de soufflet,* synchronous with the pulse, along the course of the thoracic descending aorta: it was distinctly perceptible along the left side of the spine, for the extent of about five inches upwards from the last rib. It was not possible to say how long this sound lasted; for it was only detected accidentally, and it disappeared as soon as the fluid in the pleural cavity began to diminish, as evinced by the decrease in the extent of dulness upon percussion.

(We gladly avail ourselves of the present opportunity of returning our best thanks to Dr. Hall for the first four numbers of his new Journal, and of expressing our most cordial wishes for its success. It is truly gratifying to us to receive communications from our brethren in every region of the globe, from the far West—

"Usque Auroram et Gangem;"

and we shall ever esteem it alike our pleasure and our privilege to be made the medium of converging the scattered rays of medical intelligence into one focus.)

---

### Curious Case of Simulated Paraplegia; with Remarks on this Disease.

We have received from Dr. Macloughlin of Paris a most interesting pamphlet, (2nd Edition), entitled "Consultation Medico-Legale sur quelques Signes de Paralysies vrais, et sur leur Valeur relative." The perusal of it has afforded us very true satisfaction; and we cannot but congratulate the author, as a talented physician, and a man of high and independent spirit, upon the complete success with which he has vindicated his character, not only from the malicious calumnies of a convicted impostor, but also from the unhandsome insinuations of a brother practitioner, M. Cruveilhier.

An English woman, of the name of Hardern, who had been previously convicted of simulating Ascites and Cancer of the womb by Dr. Macloughlin, applied to Dr. Mercier for a certificate stating that she was affected with a disease of the spinal-marrow, the symptoms of which she managed to feign. The Doctor was not satisfied, and he therefore declined to give any certificate. Two days after this refusal, M. Cruveilhier was called to see her; and he, believing her statements, certified that "she was affected with complete Paraplegia, accompanied with palsy of the bladder." Her object in obtaining the certificate was to entitle her to assistance from a Benevolent Society in Paris for the relief of poor English people. Dr. Macloughlin was instructed by the Committee to visit her and ascertain the truth of her statements.

"The husband invited me," says Dr. M. "to do as M. Cruveilhier had done, by inserting needles into the flesh, and by pinching the skin. At the moment when she expected that I was going to use a needle, I merely tickled her; the member bounded up under my hand, evidently possessing all its sensibility and power of motion."

M. Cruveilheir, on being made acquainted with Dr. M.'s opinion, again visited the woman; and then not only re-asserted his former diagnosis, but added that, besides a complete Hemiplegia, there was also palsy of the right arm.

The Committee of the Society, being thus placed between two very discordant opinions, referred the case to MM. Andral and Sanson. These gentlemen drew up the following report:—

"The undersigned physicians, after having attentively examined the state of Madame ———, have found as follows:—

The pupil of both eyes is sensible to light; that of the right side, however, less so than that of the left.

The movements of the tongue appear to be so much *géné*, as not to allow the patient to articulate sounds.

In a state of repose, the angles of the mouth are not on an equal level, and the same is the case when they are in action; that of the right side is always either higher or lower than the other.

The right arm and the two lower extremities are flabby and without any life. The flesh is pale and soft; that of the right arm is softer and more flabby than that of the left.

A person may doubtless, though with great difficulty, simulate both a defect of parallelism between the two angles of the mouth, and the flaccidity of the extremities; *but, what cannot be simulated, is the irregularity between the pupils and the flaccidity of the flesh.*

Now, as it has been already said, the pupil of the right side is less contracted than that of the left, and the flesh of the right arm is softer and more flabby than that of the opposite member.

We may add that different means of producing, unawares, pain in the lower extremities, produced no apparent effect upon the patient.

From the preceding facts, the undersigned physicians think it right to certify that most of the symptoms experienced by Madame ——— are real, and that they depend upon a lesion of the nervous centres.

They add that her state appears to them most painful, and well deserving interest and compassion.

ANDRAL,

Paris, le 7 Janvier, 1839. SANSON.

P.S.—As the patient admitted that she generally passed her water without difficulty, the bladder was not examined with the catheter."

In spite of this certificate, the Committee were of opinion that the woman was shamming, and accused both her and her husband of imposture before the "procureur du roi." The man was sent to prison, and Dr. Ollivier d'Angers was ordered to visit the woman and make an official report upon her case. This physician, being unacquainted with the language (English) of his patient, allowed himself at first to believe in the reality of her alleged palsy:—"je ne pus que dire, *sans l'affirmer*, qu'elle presentait les signes d'une paralysie des membres inferieurs, du membre superieur droit, et de la langue." Subsequently, however, he made a more rigid examination of the case, and candidly admitted that he had been led into error, and that "he was now convinced of the simulation of the palsy." Here we must not omit to mention that M. Ollivier did not discover any irregularity between the pupils of the two eyes, or that the muscles of the right arm were at all more flabby than those of the left one—as alleged by MM. Andral and Sanson only a week before—and yet it was upon those very two circumstances that these gentlemen chiefly based their diagnosis that the case was one of genuine palsy.

In August 1839 (eight or nine months after his first examination), Dr. Macloughlin, finding that the husband of the woman had been busy circulating various libels, in which the name of M. Cruveilhier was freely used, against him, called upon this gentleman to visit Mrs. Hardern again, and publicly to state his opinion of her case. After seeing her several times, he wrote a certificate "*qu'elle est atteinte d'une paraplegie des plus completes.*" It will be observed that no allusion is now made to the palsy of the bladder. On being applied to by Dr. M. for an explanation upon this point, M. Cruveilhier said that the state of the bladder had very little to do with the question whether there was, or was not, a *complete* Paraplegia; for he had seen many cases of *complete* Paraplegia, in which the functions of the bladder and rectum remained quite intact. Dr. M. disputed this position, contending that, "if the bladder is not paralysed, the lower extremities cannot be *completely* paralysed. The experiments of all physiologists, and the observations of all pathological writers, tend to prove this."

Dr. M. finding that the man Hardern was still continuing his calumnies and had even placarded the walls of Paris with a foul libel against him, had him brought before the police court, which, being satisfied of his guilt, condemned him to imprisonment. He appealed to the Cour Royale; and, to Dr. M.'s great surprise, who should appear in defence of the prisoner, but M. Cruveilhier? This court, however, confirmed the sentence; and straightway M. Cruveilhier opened an asylum to the woman in the Hôpital de la Charité, where he delivered several lectures upon her case with the view of proving the accuracy of his own diagnosis! In February 1840, she was still in the hospital, her case being considered by M. C. to be "an organic lesion of the upper part of the spinal marrow," producing paralysis not only of the lower extremities, urinary bladder and rectum, but also of the right side of the cranium, tongue, and right arm. Dr. Macloughlin undertook to prove the fallacy of this diagnosis before all the students of the hospital. His exposition of the various circumstances of the patient's condition is very elaborately and ably drawn out: we can, however, only allude to a few particulars. Although the woman had been bed-ridden for more than twelve-months, there was no abrasion, nor even any redness on the skin of the back and hips. The state of the anal and urethral orifices, and of the woman's linen shewed that there was no incontinence of the rectum and bladder; and when a catheter was introduced, the urine flowed out steadily in a continuous stream, for at least three or four inches beyond the end of the instrument: the urine too was *acid and contained no mucosities.* The paralytic parts had lost none of their natural heat; there was no want of cutaneous transpiration, nor was there any tendency to exfoliation of the skin; the muscles were not flabby and atrophied; the fleshy points of the fingers were not wasted; and the pulse was not weaker in the paralytic than in the sound arm.

As a matter of course, in a case of suspected simulation of the loss of power and sensation in a limb, no prudent physician would attach much importance to the mere circumstance of a patient not wincing upon the skin being pinched or a needle being thrust into the flesh; yet, strange to say, M. Cruveilhier seems to have allowed himself to be deceived by this very common trick. Dr. Macloughlin wisely paid but little attention to this point, and we need not therefore notice it. His remarks upon another subject are so good that we are tempted to give them nearly entire.

"The first means, that we employed to shew that there was a voluntary movement in the muscles of the (alleged) palsied arm, was to rest the elbow on a hard surface, the hand being placed in a *supine* position. When left to itself, the hand gradually assumed that of *pronation.* This circumstance naturally suggested that the act was partly a voluntary one; but to make this the more apparent, we adopted the following plan. The elbow still resting on the hard surface of the hand being *supine,* we laid hold of the patient's right arm with our left hand, keeping the left thumb pressed upon the radius. After a few minutes, we distinctly felt the effort made to bring this bone round into the *prone* position; and this was so forcible that we had some difficulty in preventing the bone from rotation.* When the pressure was removed, the radius gradually revolved round, until the hand was in a state of pronation. To make the experiment still more conclusive, a weight was attached to the thumb, while the hand rested on a firm surface in a supine position. If the member is not paralysed, the thumb will

* In conducting this experiment, it will be well to divert the patient's attention from his hand, by engaging him in conversation upon other topics.

then, instead of remaining extended, become ere long bent, in order the better to support the affixed weight depending from it. Such proved to be the case on the present occasion. M. Cruveilhier, notwithstanding these phenomena, persisted in asserting that the right arm was paralytic !"

To test the condition of the lower extremities, one of the best plans is to make the patient lie flat upon his back, his hips just resting on the edge of the bed, while his legs hang down without reaching the ground. They are now to be gradually raised, by the little toes being laid hold of, to the horizontal line; and a slight traction should then be made on these toes, as if the operator wished to extend the leg, keeping the patient's attention diverted, as on the former occasion. If the limb is not paralytic, it will be found that gradually it begins to feel lighter in the hands; the patellæ at the same time become more salient, and the *vasti* and *rectus* muscles of the thigh swell out and are more prominent under the skin, without however exhibiting any convulsive movement.

Now, as all these appearances were observed in the case of Mrs. Hardern, Dr. M. did not hesitate to proclaim it to be one of feigned, and not of real, palsy.

That his opinion was from the first correct, we have not a doubt; and, if we ever had, it would be removed, as a matter of course, by learning that the woman was afterwards *proved* to have signed her name with her right (the palsied) hand to several letters, six months after the date of the controversy that took place between him and M. Cruveilhier in the wards of La Charité. It would seem also that the learned Professor had called several of his colleagues into consultation upon the case, but that they declined to join him in giving the patient a fresh certificate of her palsied condition.

The woman and her husband subsequently took themselves off to Italy, and, at the beginning of the present year, they were living in Naples; although M. Cruveilhier had, four years ago, predicted her speedy dissolution in consequence of an organic disease at the base of the Brain; and had also introduced, into the 35th part of his *Anatomie Pathologique,* a short history of the case, which, he says, had been "*meconnue par d'autres confreres.*"

---

### NEW ANALYSIS OF THE SOUNDS OF THE HEART. By M. *Rouanet.*

IT is now upwards of twelve years since M. Rouanet propounded his views on this much disputed point; yet, strange to say, although these views have been canvassed by almost every subsequent writer on the subject of Cardiac Auscultation, and his theory has met with more favour than almost any other, this gentleman has never mingled in the discussion, until very recently, in drawing up a report on a work just published by M. Andry, and entitled "Manuel de Diagnostic." In this Report, M. Rouanet first examines the various objections that have been made to his theory, as well as the claims of other theories that have been proposed, and he then proceeds to give, what he considers to be, an explanation of the abnormal sounds of the Heart.

The question as to the healthy or natural sounds of the Heart has received more than thirty different solutions, all of them based upon four primary elements, either simple and isolated, or variously combined :—viz. 1, the muscular sound (myophonia); 2, the friction of the blood; 3, its shock; and 4, the clicking of the valves.

We may assume it, as sufficiently proved, that neither the muscular sound, nor the friction of the blood against the orifices of the heart, nor the shock of the columns of the blood against each other, nor that of the blood against the walls of the heart, nor yet the impulse of this organ against the thoracic parietes, can afford any satisfactory explanation of the *tictac.* The arguments adduced by M. Rouanet may be deemed conclusive against each of these hypotheses. But he would seem to be less fortunate in establishing his own views than in refuting those of others. He has proposed a very singular explanation of the metallic or silvery sound of the heart. "If," says he, "the *tragus* of the ear be smartly depressed upon the posterior wall of the external auditory passage, a portion of the air contained in this passage quickly escapes between the approximated surfaces, and produces a silvery *souffle.* What we thus obtain with so much facility by the direct action of the point of the finger, or by the percussion of the hand applied upon the ear, the heart effects in its energetic contractions, by raising the fifth intercostal space against the head of the auscultator. If, after having depressed the *tragus,* it is allowed to rise up quickly, a second *souffle* exactly resembling the preceding one, results from the re-entrance of the air into the passage of the ear."

It is at once obvious how M. Rouanet has been led to adopt this explanation. If the heart never leaves the thoracic parietes, but is always in contact with them, there can be no *shock,* only an *impulsion;* the impulsion, however strong it may be, can hardly account for the metallic sound; the cause of this sound does not reside in the thoracic parietes; it must be more superficial; and it is then that M. Rouanet imagines this depression of the *trajus* against the posterior wall of the auditory passage.

A host of objections might be urged against such an explanation. Passing by any allusion to the *gastric tinnitus,* of which the author had previously treated, and which is not, properly speaking, a metallic sound, the heart may still give out two sorts of silvery sound. One is a sort of *frôlement,* or grazing murmur, prolonged during a part of the systole, or of the diastole; the other consists in a dry and instantaneous sound. These two sounds are so very different in their nature that they cannot possibly be produced by absolutely identical conditions. M. Rouanet, who only speaks of the *frôlement,* compares it to the sound produced by the point of the finger being introduced into the auditory passage, or to that which follows the percussion of the hand applied over the ear. Now, the least experience will suffice to shew that these are dissimilar sounds; for the first resembles a kind of clear-toned *souffle* that is slightly sibilant, whereas the second is brusque, instantaneous and truly metallic. M. Rouanet believes that these two sounds are produced by an analogous mechanism, and that this consists in a rapid current of air at the entrance of the ear; he supposes that the percussion of the hand applied upon the ear depresses the tragus, and expels the air from the auditory passage, just in the same manner as the extremity of the finger does by diminishing the capacity of this passage; and that this is the cause of the metallic sound.

Now there are a thousand ways of shewing that this supposition is erroneous. For example, instead of one hand being placed flat upon the ear, let two, three, or four be applied, and slightly struck in such a manner that the shock is not felt in the vestibule of the ear; the *tragus,* certainly, will not have been 'refoulé,' and yet the sound produced will be found to be perfectly metallic. Better still, either apply your left hand over the ear, and strike the olecranon of the same side, or place your ear flat upon any part of the chest of a person, and strike at some distance, and you will always obtain the same silvery sound. In all these experiments, the sound produced is identical with that one of the two metallic sounds of the heart, which we have said is dry and instantaneous: it differs, on the contrary, totally from the other sound which we have described as having a *grazing* character, (frolement). Thus, then, the mediate or immediate shock of the thoracic parietes is really the condition which produces one of the two metallic sounds of the heart; and the depression of the *tragus* absolutely goes for nothing. But, it may be asked, can this depression be appealed to, to explain the silvery *frôlement?* We think not; for, besides that the sound, which is produced at will in this manner, has really nothing silvery in it, the *frôlement,* as well as the sound of the shock, may be heard in many circumstances where the *tragus* receives no impulsion whatsoever. Repeat all the experiments previously mentioned, substituting only for the shock a slight friction; for example, rub softly; either the hand applied upon the ear or the olecranon of the same side, and you will obtain a most decided metallic *frôlement*, so that we cannot for a moment doubt that the friction of the heart against the thoracic parietes is the cause of the grazing sound, which is occasionally perceptible in disease; and that a shock against the same parietes is the cause of the other dryer sound of which we first spoke.

M. Rouanet dwells with much emphasis on the facility with which the "dedoublement" of the physiological cardiac sounds—a phenomenon so puzzling to the theories of muscular bruit, shock, or friction—can be accounted for by his, the valvular, theory. It is quite easy to understand how—notwithstanding the most perfect synchronism in the movements of the two halves of the heart—the corresponding valves of the two sides may not, in consequence of some disturbance of their normal play, be opened and shut at the same moment of time: this circumstance alone might incline one to account for the "dedoublement" of the sounds in this way, and, in short, to admit the truth of M. Rouanet's theory *in toto*. And yet we must frankly acknowledge that we are by no means satisfied, that the cause of the first sound is fairly made out. The English experimenters agree with M. Rouanet as to the cause of the first sound, but not as to that of the second one. We know that MM. Cruveilhier and Monod found that the auriculo-ventricular valves were silent (aphones) in a very remarkable case of congenital malformation, where the heart of a new-born child was naked and exposed; and the result of our own clinical observations has been to satisfy us that these valves have little or nothing to do with the production of the cardiac sounds in a state of health. Upon this point, it must be confessed that science still stands in need of more than one element of solution.

One word more as to the explanation which M. Rouanet gives of the blowing sounds of the heart and arteries. According to him, they should all be referred to the vibration of the blood. This vibration takes place every time that the blood forms a whirling eddy, and the blood eddies in this manner whenever it passes from a narrow canal into one that is larger, provided it be propelled with great rapidity. In this simple manner, M. Rouanet explains all the varieties of the blowing sound, from the simple *souffle* to the musical *souffle*, and all the well-known caprices of the chlorotic *souffle*. The ingenuity, with which these ideas are supported, gives them a great appearance of probability; but it must be regretted that they had not been submitted to the test of experiment. More than once, it has seemed to us that a simple and easy experiment would immediately have demonstrated what the author contents himself with simply asserting. The fundamental idea itself, that of the whirling eddies of the blood considered as causes of the blowing sounds, was susceptible of this kind of demonstration. Thus many objections might have been obviated, which the mind, when left to mere reasoning, is apt to suggest. It may be asked, for example, why the blood, in entering into the ventricles, and in passing out from them, does not normally give place to a double blowing sound. "It is," says M. Rouanet, "because the sanguineous current in health is not sufficiently accelerated to render the vibrations of the blood appreciable." But what takes place in fever? What follows after quick running? Then the heart often beats 100, 110, 120, 130 times a minute, without (except in the case of organic malady or of chlorosis) the blowing sound being heard. Nevertheless we repeat that we do not absolutely reject the explanation of M. Rouanet; it even seems to us to give a better account of the phenomena than any other; we only regret that it is not based upon less disputable foundations. He will, no doubt, excuse this remark of our's, seeing that it only arises from our conviction of the great benefit that science would derive from experimental researches undertaken in this path by the ingenious author of the Valvular Theory.

M. Dechambre—the author of the preceding remarks—concludes them with the following *petite reclamation.*

" M. Rouanet, after stating that the retardation of the circulation in old people prevents certain lesions of the valves of the heart from giving rise to any blowing sounds, adds these words ; ' *Voilà pourquoi* the objections of MM. Piorry and Dechambre are without much value.' Objections to what ?—As far as regards me, I have never written a single word of controversy upon the point in question ; all that I ever did was to communicate to M. Piorry the summary of some cases in which I had found on dissection contraction of the cardiac valves, although there had been no abnormal sounds discoverable during life. These facts M. Rouanet admits as well as myself ; and, on my part, I quite agree with him that the retardation of the circulation is sufficient to prevent a valvular contraction from giving rise to the characteristic *souffle*."—*Gazette Medicale, No.* 28.

---

### M. Parchappe on the Sounds of the Heart.

During the course of last year, a Treatise, entitled " *Du Coeur, de sa Structure, et de ses Mouvemens*," was published by M. Parchappe. According to the report of the *Gazette Medicale*, the work contains a good deal of interesting matter on the Anatomy and Physiology of the Heart. We select the closing paragraph of the review.

" A fact independent of all doctrines, and one that is very important in itself, has been particularly noticed by this author. It is well known that physicians are not at all agreed among themselves upon the question, whether the normal cardiac sounds continue to be perceived, when the heart of an animal is laid bare and taken out of the chest. M. Parchappe, on this as in the other problems which he has examined, adduces not merely an opinion, but the direct result of his observations. ' I can affirm,' he says, ' that the *tic-tac*, so clearly perceived by the mediate auscultation of the heart in the rabbit, has entirely ceased to be perceptible by me every time I have applied the stethoscope to the heart when laid bare, at a moment when, its movements still continuing to be produced, the circulation of the blood through its cavities had ceased. And I can also assert that the stethoscope, applied to the heart of the rabbit taken from the chest, placed upon a table and still contracting with sufficient force to give to the ear a distinct sensation of rising or heaving, has never enabled me to discover any sound.'

We could multiply these extracts, without their ceasing to preserve the same character of conscientious and profound observation. But we must refrain ; and leave the reader free to study in the text these documents which will rank henceforth among the most useful materials for a complete anatomical and physiological history of the heart."

(*Remarks.*—After all the experiments that have been performed, and all the long-drawn, and often too most angry, controversies that have taken place, in this and other countries, on the subject of the ætiology of the normal sounds of the Heart, it must be confessed that the question still remains undecided. Our only motive for alluding to the matter at present is again (for this is not the first or second time that we have spoken out) to enter our protest against the repetition of those barbarous experiments on living animals, the performance of which is so disgraceful, upon the whole, to the medical profession. And what good have they done ?—literally none. We verily believe that there would have been much more rational views on this, as well as upon many other disputed questions in physiology, if observation had been exclusively confined to noticing the natural and spontaneous phenomena of life in health and disease, and diligently examining after death the effects which disease leaves behind. Shall we believe that any conclusion, worth knowing, can be drawn from cutting a poor animal's heart out of its body and applying a stethoscope to its surface? Away with such butcher-like philosophy!—*Rev.*)

---

ON CATARACT, AND ITS APPROPRIATE TREATMENT. By *Charles G. Guthrie,* Assistant-Surgeon to the Royal Westminster Ophthalmic Hospital. 8vo. pp. 127. 1845.

WE had hoped to have found room in the present number for a review of this and several other books which call for notice. Among these we may more especially mention "Coles on Spinal Affections, and the prone system of treating them," (a work that contains many most judicious observations), "Klenckes' Contagiosität der Eingeweidewurmer," "Simon's Animal Chemistry," &c. &c.

All that we can now do is very briefly to call attention to Mr. Guthrie's work, which, although it might easily have been much improved, may be read with very considerable advantage by medical men.

*Symptoms of Cataract.*

"When idiopathic cataract is about to take place, the patient usually complains of a little weakness of sight, or rather, that it becomes indistinct, or confused, and a greater degree of attention is required to fix and distinguish objects accurately. They appear as if seen through a thin mist, or a semi-transparent turbid fluid, or through a glass which is dirty or dull. This indistinctness of vision is constant. No change or motion of the head, or rubbing of the eye improves the sight, whilst the patient remains exposed to the same degree of light; but if the room be darkened, the vision is in some cases considerably improved. The patient is frequently seen to place his hand on a line with the eyebrow, or what is termed shading the eye from the light, under which circumstances the pupil is dilated, and objects are seen mo[illegible] distinctly. The improvement takes place only in persons who are suffering from lenticular cataract, and particularly in those in whom the opacity commences in the centre of the lens. The advantage is gradually lost as the pupil contracts, or is exposed to a greater light; in which case he is obliged to bring every object nearer to his eye, whilst it is often more readily seen from one side, than when placed in the axis of vission." P. 7.

There is often no little difficulty in *distinguishing incipient Cataract,* before the opacity can be perceived, *from amaurotic weakness.*

"Vision, in incipient amaurosis, is not improved by the use of spectacles, or by the application of belladonna, which rather increases the defect, and the sufferer usually sees better under an increase of light; for instance at noon-day, when the rays of light are brighter, or if he reads by candle-light, he throws a strong light upon the book, and even then brings it nearer to the eye. Incipient amaurosis is sometimes, however, accompanied by increase of sensibility of the external parts, and then a strong light distresses the patient, who can only see when it is carefully moderated. The flame of a bright lamp thus seen, is usually more or less coloured or variegated, blue, yellow, or red, like the rays observable in a rainbow, or more broken and confused, whilst in incipient cataract, the flame of the lamp seems to be surrounded only by a whiter mist of milder light, and which appears to make objects larger than they really are. Flashes of light, or white and brilliant circles, or luminous spots, are equally indicative of amaurosis, more particularly if seen when the eye is closed during the night, and the patients in whom these symptoms occur, usually suffer from headache and pain, especially in the forehead and over the eye, or from giddiness, symptoms which are not common accompaniments of incipient cataract." P. 25.

Many medical men believe that the Iris is always motionless—at least to a considerable degree—in complete Amaurosis: this is far from being uniformly the case. In many instances, where vision has been entirely lost from a defect in the retina or in the optic nerve, the pupils will be found to contract and dilate under the influence of light. The reverse of this is also true; for the iris may lose its contractility, while the retina retains its sensibility. Mr. Guthrie says that " the healthy state of the Iris is *generally* a good, although not an unerring, index of the healthy state of the Retina; whilst a diseased state or loss of function of the Iris by no means indicates, although it may lead to a suspicion of, a diseased state of the Retina."

